# S100A8 in Serum, Urine, and Saliva as a Potential Biomarker for Systemic Lupus Erythematosus

**DOI:** 10.3389/fimmu.2022.886209

**Published:** 2022-04-22

**Authors:** Ji-Won Kim, Ju-Yang Jung, Sang-Won Lee, Wook-Young Baek, Hyoun-Ah Kim, Chang-Hee Suh

**Affiliations:** ^1^ Department of Rheumatology, Ajou University School of Medicine, Suwon, South Korea; ^2^ Department of Molecular Science and Technology, Ajou University, Suwon, South Korea

**Keywords:** S100A8, systemic lupus erythematosus, biomarkers, biofluids, disease activity

## Abstract

**Objectives:**

This study aimed to elucidate the potential of serum, urine, and saliva S100 calcium-binding protein A8 protein (S100A8) levels as biomarkers for systemic lupus erythematosus (SLE).

**Methods:**

Serum, urine, and saliva samples were obtained from 249 patients with SLE from the Ajou lupus cohort and 52 age- and sex-matched healthy controls (HCs). The concentrations of S100A8 were quantified using an ELISA, and a receiver operating characteristic curve was used to analyze whether they may be used as biomarkers for diagnosing SLE.

**Results:**

Among 249 SLE patients included in our study, the mean SLE disease activity index (SLEDAI)-2K was 7.16 ± 5.61, and the number of patients with lupus flare was 11. Patients with SLE showed a 2.7-fold increase in serum S100A8 levels compared with that in HCs (1,890.6 vs. 709 pg/ml, p < 0.001). In urine and saliva, the average S100A8 levels were significantly higher in patients with SLE compared with those in HCs (urine, 2,029.4 vs. 1,096.7 pg/ml, p = 0.001; saliva, 290,496.3 vs. 47,742 pg/ml, p < 0.001). For SLE diagnosis, the area under the receiver operating characteristic curve was 0.831 for serum S100A8 (95% CI, 0.765–0.897), 0.751 for urine S100A8 (95% CI, 0.648–0.854), and 0.729 for salivary S100A8 (95% CI, 0.646–0.812). Pearson’s correlation analysis showed that S100A8 in serum, urine, and saliva was significantly associated with the SLEDAI (r = 0.267, p < 0.001; r = 0.274, p < 0.001; and r = 0.629, p < 0.001, respectively). Among the clinical manifestations, nephritis was the most influential factor related to SLE in the concentration of S100A8 in serum, urine, and saliva.

**Conclusion:**

This is the first study to show that the expression of S100A8 in serum, urine, and saliva is significantly higher in patients with SLE than in HCs and is associated with disease activity markers. Therefore, we suggest that S100A8 protein could be a potential biomarker for SLE.

## Introduction

Systemic lupus erythematosus (SLE) is a chronic autoimmune disease characterized by the production of autoantibodies owing to the loss of immunological tolerance to autoantigens caused by genetic, hormonal, and environmental factors (1). Dysregulation of the immune system attacks healthy cells and tissues, causing inflammation in multiple organs, including the skin, joints, and kidneys, and results in a wide range of clinical manifestations (2). The classification and diagnosis of SLE are complex and difficult because of the nature of this multisystemic disease and an incomplete understanding of its pathophysiology (3). Although classification criteria that combine diverse clinical symptoms and supportive serologic abnormalities are used to diagnose SLE, the diagnosis does not rely solely on the fulfillment of the classification criteria, and the final diagnosis is left to the clinicians’ judgment (4). However, it is challenging to make a prompt diagnosis even for an experienced rheumatologist because of the heterogeneity in both the expression of various clinical symptoms and profiles of autoantibodies. Therefore, serological biomarkers that can meet the currently unmet diagnostic needs are required.

Various biomarkers have been proposed in SLE to overcome the difficulty of diagnosis due to the heterogeneity of the manifestations, many of which have been well validated, and some of which are being used in clinical practice (5). One biomarker with established clinical significance in SLE is S100 calcium-binding protein A8 protein (S100A8) (6). S100A8 is a Ca^2+^-binding protein belonging to the S100 family that is released from neutrophils as part of neutrophil extracellular traps during an inflammatory response (7). Although mainly known in the heterodimer of S100A8/A9, S100A8 also acts as a damage-associated molecular pattern molecule after release and accumulates in the blood and body compartments, as it is an important regulator of inflammation that promotes the function of innate immune cells through interaction with toll-like receptor 4 and the receptor of advanced glycation end products, which are members of the immunoglobulin superfamily of cell surface molecules (8–10). There is growing experimental and clinical evidence that serum S100A8 levels are higher in patients with SLE than in healthy controls (HCs) and are associated with disease activity, glomerulonephritis, and anti-double-stranded DNA (dsDNA) antibodies (Ab) (11–13). However, the increased serum S100A8 level may be insufficient in its role as an SLE-specific biomarker, given that it is also observed in many inflammatory diseases such as rheumatoid arthritis and inflammatory bowel disease (14, 15).

There is a growing interest in combining biomarkers to improve the predictive value to obtain an accurate and early diagnosis of the disease. Considering the difficulty in finding candidate biomarkers and the high cost of obtaining them, it is preferable to collect and combine specific biomarkers from several biofluids rather than to use multiple serum biomarkers (16). Among biofluids, researchers mainly use serum, saliva, urine, and tears because of factors such as ease of access, minimization of invasive sampling, and availability of multiple sampling (17). S100A8 protein has also been analyzed as a biomarker in several biofluids; however, there is no comparative evidence for S100A8 homodimer in the saliva and urine of patients with SLE. Hence, in this study, we hypothesized that S100A8 homodimer could be useful as a biomarker for identifying the onset of SLE, and to prove this, we compared the screening ability of S100A8 homodimer in serum, urine, and saliva from patients with SLE and HCs.

## Materials and Methods

### Study Population and Clinical Assessments

This study enrolled 249 patients with SLE from the Ajou lupus cohort at the Department of Rheumatology, Ajou University Hospital, Republic of Korea. SLE diagnosis was based on the 1997 American College of Rheumatology (ACR) criteria or the 2012 Systemic Lupus International Collaborating Clinics (SLICC) classification criteria (18, 19). Patients with other autoimmune diseases, such as Sjogren’s syndrome, rheumatoid arthritis, and systemic sclerosis, were excluded. Informed consent was obtained from each patient prior to sample collection, and 249 serum and urine samples and 100 saliva samples were collected, excluding patients who did not want to participate. Demographic, clinical, therapeutic, and laboratory data related to SLE were gathered from the patients’ medical records.

In our center, anti-double-stranded DNA (anti-dsDNA) antibodies were assayed using Anti-dsDNA kit (Trinity Biotech, Bray, Ireland), and anti-dsDNA values >7 IU/ml were defined as abnormal. Complement 3 (C3) and complement 4 (C4) levels were measured on Cobas (Roche Diagnostics, Basel, Switzerland), with a normal range of C3 of 90–180 and C4 of 10–40 mg/dl.

Disease activity was assessed using the SLE disease activity index (SLEDAI)-2K at the time of sample collection (20). Lupus flares were defined as a ≥3 point increase in SELENA-SLEDAI according to SELENA-SLEDAI Flare Index (21). Damage was determined by the SLICC/ACR damage index (SDI) score calculated based on 12 different organ damages that occurred after diagnosis of SLE, and significant organ damage was defined as SDI ≥ 1 (22). Fifty-two age- and sex-matched HCs were recruited from the same region. The study protocol was reviewed and approved by the Institutional Review Board of Ajou University Hospital (AJIRB-BMR-SMP-19-403).

### ELISA for S100A8 Proteins

Venous blood, unstimulated saliva, and urine were collected from patients with SLE and HCs, and the serum was immediately centrifuged at 15,928 relative centrifugal force (RCF) and saliva at 1,763 RCF for 10 min. The supernatant was collected and stored at −80°C until further analysis. Before ELISA was conducted, frozen serum, urine, and saliva samples were thawed and then diluted 1:100 in phosphate-buffered saline. S100A8 homodimer concentrations were measured using a commercially available ELISA kit (MBS2022637; MyBioSource, San Diego, CA, USA) for serum and urine. S100A8 homodimer concentrations in saliva were evaluated using the Human S100A8 ELISA kit (R&D Systems, Minneapolis, MN, USA) (cat. No. DY4570-05). All materials were supplied with the kit, and the test was performed according to the manufacturer’s instructions.

### Statistical Analysis

To determine the baseline differences in populations, Student’s *t*-test or Mann–Whitney U test was performed for continuous variables, and the chi-square test or Fisher’s exact test was performed for categorical variables. The results were expressed as mean ± SD, and all statistical significance was set at a p-value <0.05. By analyzing the area under the curve (AUC) of the receiver operating characteristic (ROC) curve, we established the utility of S100A8 levels in serum, urine, and saliva as a diagnostic marker to distinguish patients with SLE from HCs. Youden’s index (calculated as sensitivity + specificity − 1) was used to determine the cutoff values for S100A8 levels. We also calculated sensitivity, specificity, positive predictive value (PPV), and negative predictive value (NPV). Pearson’s correlation analysis was conducted to evaluate the relationship between S100A8 levels and the disease activity index. All statistical analyses were performed using SPSS software (version 25.0; IBM Corporation, Armonk, NY, USA).

## Results

### Baseline Clinical Characteristics of Patients With Systemic Lupus Erythematosus and Healthy Controls

The baseline patient characteristics are presented in **Table 1**. The mean age of patients with SLE was 42.1 ± 11.2 years, and 95.2% were female (no significant difference from the HCs, data not shown). The mean disease duration of SLE was 98.8 ± 73.8 months, the mean SLEDAI-2K was 7.16 ± 5.61, and the number of patients with lupus flare was 11. In patients with SLE, the most common clinical symptom was arthritis (53.8%); a total of 73 patients (29.3%) had lupus nephritis, of which 45 (18.1%) patients had >500 mg/day of urine protein/creatinine ratio (UPCR). Laboratory findings were positive for antinuclear antibody (ANA) in all cases except for seven, and anti-dsDNA Ab was positive in 94 (38.8%), anti-Sm Ab was positive in 28 (11.2%), and antiphospholipid Ab (aPL) was positive in 75 (30.1%) patients. Nearly half of the patients (49.4%) had at least one abnormally low C3 (<90 mg/dl) or C4 (<10 mg/dl) level. The majority of the patients (97.2%) were receiving hydroxychloroquine, and 176 (70.7%) were receiving corticosteroids, with a mean dose of 4.43 ± 6.34 mg. Among the patients with SLE, 93 were taking immunosuppressants, with calcineurin inhibitors being the most common, followed by mycophenolate mofetil, azathioprine, and cyclophosphamide.

**Table 1:** Demographic and clinical characteristics of patients with systemic lupus erythematosus (SLE) and healthy controls.

Variable	SLE (N = 249)
Age, years	42.1 ± 11.2
Female, no. (%)	237 (95.2)
Disease duration, months	98.8 ± 73.8
Alcohol, no. (%)	72 (41.4)
Smoking, no. (%)	21 (8.4)
Clinical manifestations	
Mucocutaneous, no. (%)	111 (44.6)
Arthritis, no. (%)	134 (53.8)
Nephritis, no. (%)	73 (29.3)
Serositis, no. (%)	10 (4)
Hematologic, no. (%)	73 (29.3)
Central nervous system, no. (%)	5 (2)
Laboratory finding	
Leukocyte,/μl (normal range 3,400–10,600)	4,929.4 ± 2,252.4
Lymphocyte,/μl (normal range 1,600–4,900)	1,419.2 ± 606.5
Platelets, ×10^3^/μl (normal range 134–387)	215.1 ± 69.1
ESR, mm/h (normal range 0–25)	15.5 ± 13.6
Complement 3, mg/dl (normal range 90–180)	88.6 ± 25.3
Complement 4, mg/dl (normal range 10–40)	18.2 ± 9.1
Anti-ds DNA (IU/ml) (normal range 0–7)	42.4 ± 111.2
Immunologic finding	
ANA positivity, no. (%)	242 (97.2)
Anti-ds DNA Ab positivity, no. (%)	94 (38.8)
Anti-Sm Ab positivity, no. (%)	28 (11.2)
aPL positivity, no. (%)	75 (30.1)
Low complements (C3 < 90 mg/dl or C4 < 10 mg/dl), no. (%)	123 (49.4)
Urinalysis	
Proteinuria (mg/day)	0.38 ± 0.85
Proteinuria > 0.5 g/day, no. (%)	45 (18.1)
Renal histology (ISN/RPS classification), no. (%)	73 (29.3)
Class II	3 (4.1)
Class III	9 (12.3)
Class IV	29 (39.7)
Class V	15 (20.5)
Class III+V	7 (9.6)
Class IV+V	10 (13.7)
SLEDAI-2K	7.16 ± 5.61
Recent SLE flare, no. (%)	11 (4.4)
SDI score ≥1, no. (%)	27 (10.8)
Treatment	
Hydroxychloroquine, no. (%)	242 (97.2)
NSAIDs, no. (%)	82 (32.9)
GCs, no. (%)	176 (70.7)
Mean GC dose, mg/day (prednisolone-equivalent)	4.43 ± 6.34
Cumulative GC dose, g (prednisolone-equivalent)	11.1 ± 13.3
Immunosuppressants no. (%)	
Azathioprine, no. (%)	27 (10.8)
Mycophenolate mofetil, no. (%)	52 (20.9)
Cyclophosphamide, no. (%)	18 (7.2)
Calcineurin inhibitor, no. (%)	53 (21.3)
ACE inhibitor or ARB, no. (%)	52 (20.9)
Vitamin D, no. (%)	190 (76.3)

Antiphospholipid antibody positivity included persistently positive (>12 weeks positivity) of at least one lupus anticoagulant, anticardiolipin, or anti-beta-2 glycoprotein I IgG or IgM.

SLE, systemic lupus erythematosus; ESR, erythrocyte sedimentation rate; ANA, antinuclear antibody; dsDNA, double-strand deoxyribonucleic acid; Ab, antibody; Sm, Smith; aPL, antiphospholipid antibodies; C3, complement 3; C4, complement 4; ISN/RPS, International Society of Nephrology and the Renal Pathology Society; SLEDAI-2K, SLE disease activity index 2000; SDI, Systemic Lupus International Collaborating Clinics/American College of Rheumatology Damage Index; NSAIDs, non-steroidal anti-inflammatory drugs; GC, glucocorticoid; ACE, angiotensin-converting enzyme; ARB, angiotensin receptor blocker; NA, not applicable.

### Concentration of S100A8 in Biofluids in Patients With Systemic Lupus Erythematosus and Healthy Controls

**Figure 1:**
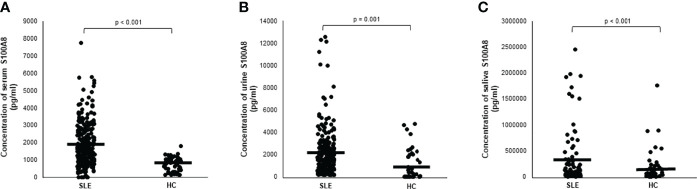
**(A)** Serum level of S100A8 in patients with systemic lupus erythematosus (SLE). **(B)** Urine level of S100A8 in patients with SLE. **(C)** Salivary level of S100A8 in patients with SLE. Central horizontal bar indicates mean value. Statistical analyses were conducted using Mann–Whitney U test.

As shown in **Figure 1A**, the serum S100A8 levels were significantly higher in patients with SLE than in HCs (1,890.6 ± 1,254.7 vs. 709 ± 413 pg/ml, p < 0.001). **Figures 1B, C** show elevated urine and saliva concentrations of S100A8 in patients with SLE compared with those in HCs (2,029.4 ± 2,251.4 vs. 1,096.7 ± 1,422.8 pg/ml, p = 0.001; and 290,496.3 ± 513,156.5 vs. 47,742.1 ± 60,875.7 pg/ml; p < 0.001, respectively).

### Receiver Operating Characteristic Curves for the Diagnosis of Systemic Lupus Erythematosus of S100A8 Levels in Saliva, Urine, and Serum

**Figure 2:**
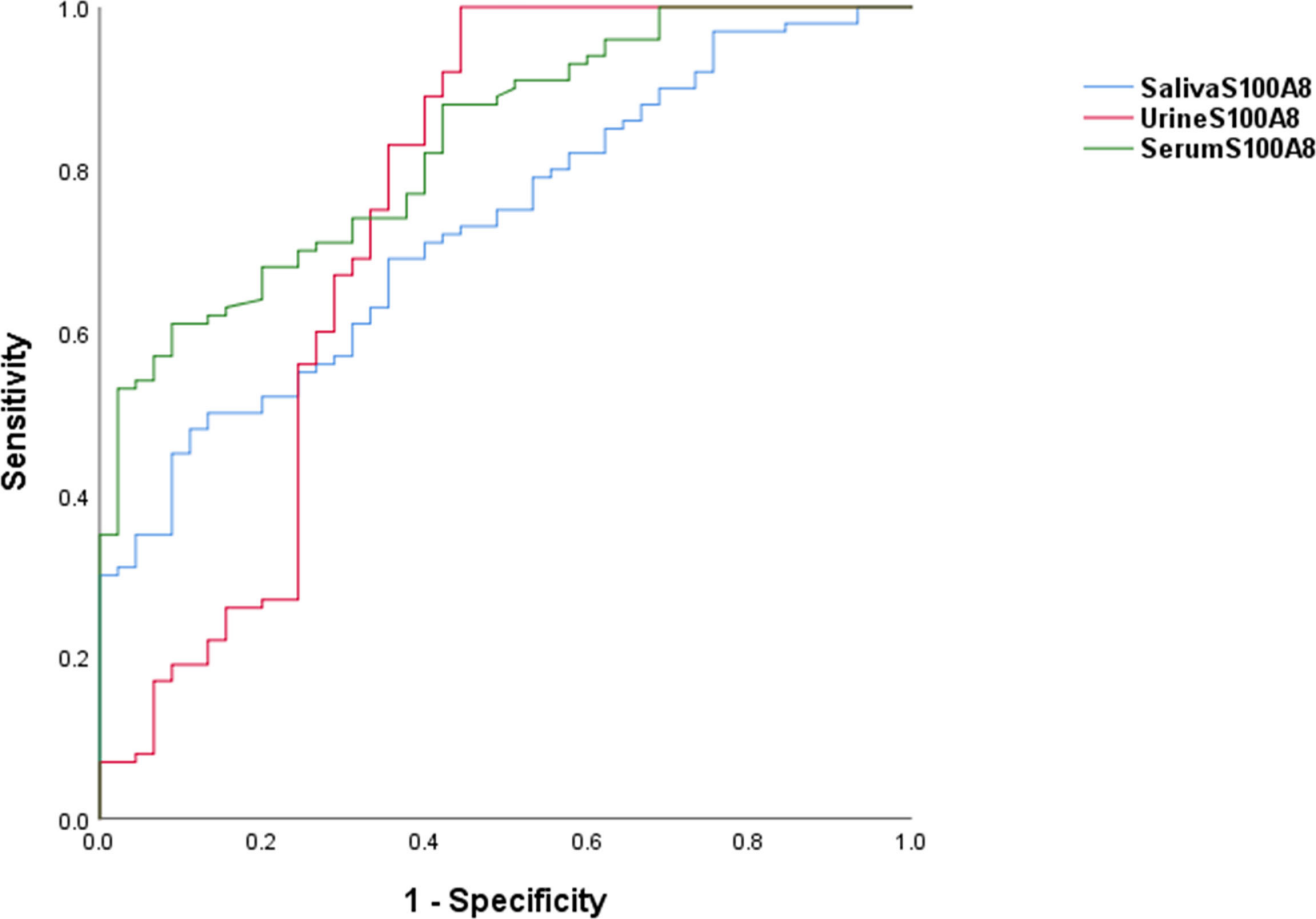
Receiver operating characteristic curves associated with the diagnostic utility of S100A8 in serum, urine, and saliva. For SLE diagnosis, the AUC was 0.831 for the serum S100A8 (95% CI, 0.765–0.897), 0.751 for the urine S100A8 (95% CI, 0.648–0.854), and 0.729 for the salivary S100A8 (95% CI, 0.646–0.812). AUC, area under the receiver operating characteristic curve; SLE, systemic lupus erythematosus.

The ROC curves for serum, urine, and salivary S100A8 levels for discriminating SLE are shown in **Figure 2**. The AUC values for serum, urine, and salivary S100A8 levels were 0.831 for serum S100A8 (95% CI, 0.765–0.897), 0.751 for urine S100A8 (95% CI, 0.648–0.854), and 0.729 for salivary S100A8 (95% CI, 0.646–0.812), with optimal cutoff values of 1,055, 512.5, and 80,269.5 pg/ml, respectively. The diagnostic ability characteristics of the biomarkers, including sensitivity, specificity, PPV, and NPV, are presented in **Table 2**. Of the three biofluid biomarkers, urine S100A8 showed the highest specificity (0.99) with the lowest sensitivity (0.556), and serum and salivary S100A8 showed both higher specificity (0.911) and lower sensitivity (0. 61 and 0.52, respectively) than urine S100A8. At the cutoff value of each biomarker, the highest PPV was 95.7% in serum, and the highest NPV was 80.6% in urine.

**Table 2:** Utility of S100A8 levels in serum, urine, and saliva for diagnosing SLE.

Variable	AUC	p-Value	Cut-off	Sensitivity	Specificity	PPV	NPV
Serum S100A8 (pg/ml)	0.831	<0.001	1055	61%	91.1%	95.7%	37.9%
Urine S100A8 (pg/ml)	0.751	<0.001	512.5	99%	55.6%	63.9%	80.6%
Salivary S100A8 (pg/ml)	0.729	<0.001	80,269.5	52%	91.1%	87.3%	46.4%

SLE, systemic lupus erythematosus; AUC, area under the curve; PPV, positive predictive value; NPV, negative predictive value.

### Correlations of S100A8 Levels With Disease Activity Index and Clinical Manifestations of Systemic Lupus Erythematosus

**Figure 3:**
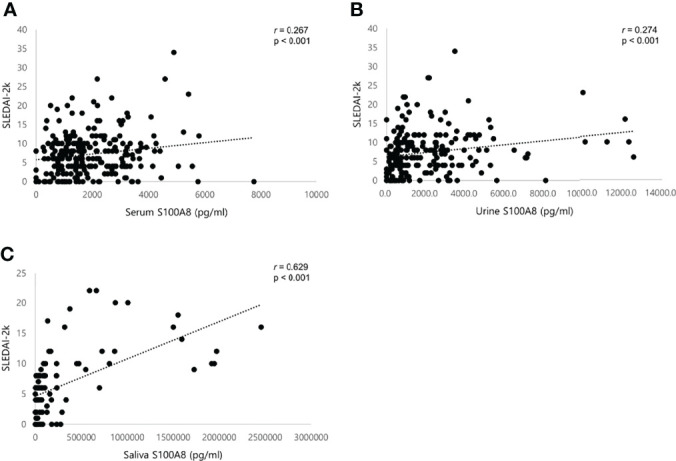
Scatter plots showing positive correlations between S100A8 and SLEDAI-2K in patients with SLE. **(A)** Serum S100A8. **(B)** Urine S100A8. **(C)** Salivary S100A8. SLEDAI, systemic lupus erythematosus (SLE) disease activity index. Statistical analyses were conducted using Pearson’s correlation analyses.

Using Pearson’s correlation, we confirmed that the S100A8 levels in serum, urine, and saliva were correlated with clinical indices explaining disease activity in SLE (**Table 3**). Serum S100A8 concentrations were positively correlated with erythrocyte sedimentation rate (ESR) (r = 0.125, p = 0.006), anti-dsDNA Ab (r = 0.204, p = 0.001), and UPCR (r = 0.127, p = 0.014) and negatively correlated with complement 3 (r = −0.205, p = 0.001). Similarly, urine S100A8 concentrations were positively correlated with anti-dsDNA Ab (r = 0.167, p = 0.012) and UPCR (r = 0.177, p = 0.018) and negatively correlated with hemoglobin (r = −0.279, p < 0.001) and complement 4 (r = −0.139, p = 0.037). ESR (r = 0.139, p = 0.043) and anti-dsDNA Ab (r = 0.179, p = 0.009) were also positively correlated with salivary S100A8 levels. No significant correlation was found between S100A8 levels and the other indices, including leukocytes, lymphocytes, and platelets. There was a significant positive correlation between the SLEDAI-2K and all biofluid biomarkers (serum, r = 0.267, p < 0.001; urine, r = 0.274, p < 0.001; saliva, r = 0.629, p < 0.001; **Figures 3A−C**).

**Table 3:** Correlation between S100A8 level and disease activity markers in patients with SLE.

Disease activity markers	Correlation coefficient, r (p-value)		
	Serum S100A8	Urine S100A8	Salivary S100A8
Leukocyte	0.072 (0.095)	−0.008 (0.91)	0.071 (0.299)
Lymphocyte	0.002 (0.959)	−0.081(0.229)	−0.051 (0.459)
Hemoglobin	−0.017 (0.695)	−0.279 (**<0.001**)	−0.09 (0.192)
Platelet	0.000 (0.248)	−0.135 (0.179)	−0.111 (0.105)
ESR	0.125 (**0.006**)	0.183 (0.006)	0.139 (**0.043**)
Complement 3	−0.205 (**0.001**)	−0.104 (0.119)	−0.107 (0.119)
Complement 4	−0.107 (0.094)	−0.139 (**0.037**)	−0.118 (0.084)
Anti-ds DNA Ab	0.204 (**0.001**)	0.167 (**0.012**)	0.179 (**0.009**)
UPCR	0.127 (**0.014**)	0.177 (**0.018**)	0.012 (0.864)
SLEDAI	0.267 (**<0.001**)	0.274 (**<0.001**)	0.629 (**<0.001**)

Bold values indicate significant p-values.

SLE, systemic lupus erythematosus; ESR, erythrocyte sedimentation rate; dsDNA, double-strand deoxyribonucleic acid; Ab, antibody; UPCR, urine protein/creatinine ratio; SLEDAI, SLE disease activity index.

We further compared the concentrations of S100A8 in serum, urine, and saliva according to clinical manifestations, but the association between clinical manifestations and S100A8 levels was different for each biofluid (**Table 4**). The only significant difference in S100A8 levels in the serum, urine, and saliva was nephritis (serum, p = 0.012; urine, p = 0.015; and saliva, p = 0.003). There was no significant difference in the results of further analysis on the levels of S100A8 according to the lupus nephritis classification. Serum S100A8 levels differed according to fever and central nervous system (CNS) involvement (p < 0.001 and p = 0.049, respectively), and urine and salivary S100A8 showed significant differences in arthritis and malar rash, respectively (p = 0.038 and p = 0.018, respectively). Symptoms of CNS lupus included seizures, headache, and vasculitis, among which three had seizures, and one patient each had headache and vasculitis. We stratified patients with SLE into low disease activity (SLEDAI < 6) and high disease activity (SLEDAI ≥ 6) groups, and S100A8 levels in all biofluids were significantly higher in the high disease activity group (serum, 2,052.6 ± 1,326.8 vs. 1,659 ± 1,107.9 pg/ml, p = 0.011; urine, 2,231.5 ± 2,396.6 vs. 1,638 ± 1,483.8 pg/ml, p = 0.02; and saliva, 487,263 ± 640,283.6 vs. 68,610.5 ± 91,553.7 pg/ml, p < 0.001). Furthermore, in patients with an SDI score ≥1 indicative of chronic damage, S100A8 in serum and saliva was higher than that of no damage.

**Table 4:** Comparison of serum, urine, and salivary S100A8 levels according to clinical manifestations in patients with SLE.

Manifestations^a^	Serum S100A8	p-Value	Urine S100A8	p-Value	Salivary S100A8	p-Value
Fever						
(+) = 11 (9)	3,198.4 ± 1,878.4	**<0.001**	1,586.4 ± 1,660.2	0.649	454,849.9 ± 528,660.4	0.316
(−) = 238 (91)	1,830.1 ± 1,189.7		1,954.7 ± 2,259.3		274,241.8 ± 511,714.2	
Oral ulcer						
(+) = 43 (19)	1,815.8 ± 1,223.4	0.668	1,892 ± 2,736.2	0.881	222,365 ± 471,954.7	0.523
(−) = 206 (81)	1,906.2 ± 1,263.5		1,708.9 ± 1289.6		306,477.8 ± 523,824.9	
Malar rash						
(+) = 36 (28)	2,004.2 ± 1,397.1	0.558	2494.3 ± 2860.3	0.155	521,816.2 ± 631,566.5	**0.018**
(−) = 213 (72)	1,871.4 ± 1,231.5		1860.3 ± 2129.4		200,538.6 ± 431,490.6	
Photosensitivity						
(+) = 13 (13)	2,368.7 ± 1,072.5	0.159	1,160.6 ± 1,414.2	0.236	491,065.2 ± 830,429.8	0.42
(−) = 236 (87)	1,864.2 ± 1,260.6		1,981.6 ± 2,268.2		265,009 ± 449,282.8	
Arthritis						
(+) = 134 (61)	1,972.4 ± 1,281.1	0.268	2,226.6 ± 2,503.5	**0.038**	253,206.3 ± 471,825.1	0.366
(−) = 115 (39)	1,795.2 ± 1,221.8		1,619.1 ± 1,855.2		348,821.8 ± 573,424.6	
Alopecia						
(+) = 44 (29)	1,881.5 ± 1,139.4	0.947	2,026.1 ± 2,423.3	0.737	447,309.9 ± 673,524.9	0.109
(−) = 205 (71)	1,893.7 ± 1,295.1		1,911.8 ± 2,177.1		226,445.7 ± 420,262.3	
Nephritis						
(+) = 73 (29)	2,240.7 ± 1,483.4	**0.012**	2,489.6 ± 2,196.6	**0.015**	606,700.8 ± 727,196.3	**0.003**
(−) = 176 (71)	1,745.3 ± 1,119.5		1,782.1 ± 2,018.2		161,313.8 ± 320,203.3	
Serositis						
(+) = 10 (6)	2,313.1 ± 1,609.9	0.278	2,555.4 ± 3,186.4	0.403	460,210 ± 727,252.3	0.406
(−) = 239 (94)	1,872.9 ± 1,238.8		1,916.2 ± 2,197.7		279,663.6 ± 499,906.5	
CNS involvement						
(+) = 5 (1)	2,984.5 ± 1,730.3	**0.049**	1,285.3 ± 1,484	0.509	868,505.5 ± 1,214,124.3	0.617
(−) = 244 (114)	1,868.1 ± 1,237.8		1,956.5 ± 2,253.3		278,700.2 ± 496,521	
High disease activity (SLEDAI >6)						
(+) = 147 (53)	2,052.6 ± 1,326.8	**0.011**	2,231.5 ± 2,396.6	**0.02**	487,263 ± 640,283.6	**<0.001**
(−) = 102 (47)	1,659 ± 1,107.9		1,638 ± 1,483.8		68,610.5 ± 91,533.7	
SDI score ≥1						
(+) =27 (14)	2,413.2 ± 1,413.6	**0.022**	1,914.1 ± 1,389	0.845	773,569.4 ± 742,893.9	**0.015**
(−) = 222 (86)	1,827 ± 1,222.3		1,999.3 ± 2,162.5		211,856.5 ± 421,428.5	

Bold values indicate significant p-values.

SLE, systemic lupus erythematosus; CNS, central nervous system; SLEDAI, SLE disease activity index; SDI, Systemic Lupus International Collaborating Clinics/American College of Rheumatology Damage Index.

a The number of patients in S100A8 is in parentheses.

## Discussion

The ability to identify SLE early through a specific sample is particularly critical, as a definitive diagnosis cannot be provided with a single laboratory indication and is difficult to distinguish from other diseases, such as infection and malignancies, due to complex clinical symptoms. Early detection of SLE usually has a significant impact on prognosis through prompt and appropriate treatment (23). Many research groups have proposed various diagnostic markers for SLE; however, early diagnosis of SLE remains challenging (24–26).

Increased serum levels of myeloid calcium-binding proteins in connective tissue diseases, including SLE, were first described in 1990. Since then, S100 proteins have been reported to be upregulated in various inflammatory diseases and malignancies by being involved in regulating cell proliferation and transcriptional factor activity (7, 12, 27, 28). S100A8 (also known as calgranulin A or myeloid-related protein-8), which belongs to the S100 family, forms the calgranulin subfamily, a group of proteins that play a crucial role in the regulation of inflammatory processes, together with S100A9 (also known as calgranulin B or myeloid-related protein-14) and S100A12 (also known as calgranulin C), and acts as a homodimer, heterodimer, or heterotetramer with S100A9 to exert biological functions (29). The S100A8/A9 heterodimer, the dominant form in serum, has been observed in patients with SLE as well as in those with cardiovascular disease in SLE and active lupus nephritis and can predict responses to treatment of SLE (6, 11, 13, 30, 31). Although it is not clear whether the small amounts of S100A8 homodimers are comparable to S100A8/A9 heterodimers *in vivo*, the role of S100A8 may also be evident, given that mortality occurred in the early stages of development only in S100A8 target-destroyed mice, whereas S100A9-deficient mice were viable and fertile (32–34). Therefore, the diagnostic values of S100A8 levels in the serum, urine, and saliva were evaluated and compared in the present study.

This is the first report comparing the diagnostic efficacy of S100A8 for SLE diagnosis, and it is novel that S100A8 in urine and saliva, as well as serum, were used. In this study, the mean S100A8 levels in the serum, urine, and saliva were significantly higher in patients with SLE than in HCs. There have been no previous studies comparing patients with SLE with HCs using the homodimer of S100A8; however, a study of 37 patients with SLE showed a significant decrease in serum S100A8 levels after treatment (35). According to the AUC results, the ability of serum S100A8 to diagnose SLE was good (AUC = 0.831), and that of urine and salivary S100A8 was fair (AUC = 0.751 and 0.729, respectively). Our data demonstrated that S100A8 was superior to previous studies evaluating the diagnostic biomarker ability of S100 proteins (S100A4, S100A8/A9, and S100A12) in serum and urine for SLE (6, 13). Furthermore, combining serum S100A8 with high specificity (91.1%) and PPV (95.7%), and urine S100A8 with high sensitivity (99%) and NPV (80.6%) further increased diagnostic accuracy (data not shown).

Salivary S100A8 has been identified as a potential diagnostic biomarker for oral cavity infection or oral cancer; however, to date, no data are available regarding S100 protein expression in the saliva of patients with SLE (36, 37). In Sjogren’s syndrome affecting exocrine glands, especially salivary and lacrimal glands, S100A8/A9 has been identified as a biomarker (38). S100A8/A9 levels in saliva have also been found to be higher in patients with systemic sclerosis and inflammatory bowel disease than in HCs (39, 40). In our study, salivary S100A8 concentrations in patients with SLE were significantly higher than those in HCs, and there was also a correlation with clinical indices reflecting disease activity, such as ESR and anti-dsDNA Ab. Salivary S100A8 has a high specificity of 0.911 despite its low diagnostic accuracy (AUC = 0.729; sensitivity, 0.52) compared with serum and urine, is non-invasive, and has the advantages of simple access and storage, making it an inexpensive screening tool.

Another important aspect of this study is that we also analyzed the relationship between disease activity and S100A8 levels in serum, urine, and saliva. The results showed that high S100A8 expression was correlated with low hemoglobin, high ESR, low complement, high anti-dsDNA Ab, and high proteinuria, similar to previous studies (12, 13). In addition, we found a statistically significant positive correlation between the expression of candidate biomarkers and the SLEDAI, one of the most popular and widely used indices for evaluating disease activity in SLE. All biomarkers have been proven to have an obvious association with SLEDAI; in particular, salivary S100A8 had a moderate positive correlation. Most of the recently published studies using serum and urine S100A8 reported that S100A8 concentrations increased as the disease activity of SLE increased (13, 41, 42), and only in a few studies were they not relevant (6). Considering its association with disease activity, S100A8 in serum, urine, and saliva may be efficient in detecting and monitoring disease progression in addition to diagnostic purposes.

We further investigated the correlation between clinical manifestations and S100A8 levels in serum, urine, and saliva. As expected, our data indicate a significant increase in urine S100A8 levels in lupus nephritis compared with extrarenal SLE, and intriguingly, S100A8 in serum and saliva also showed a significant increase in patients with lupus nephritis. Similar to the increase in urine S1008 in lupus nephritis in this study, another study with neuropsychiatric SLE showed an increase in S100 protein in cerebrospinal (CSF) fluid, whereas salivary S100A8 was not found to have a clear correlation with oral manifestations (43). Another noteworthy point is that the level of S100A8 was high in the serum of patients with CNS lupus, but since the sample size was small, additional studies will be needed to assert the diagnostic utility of S100A8 in CNS lupus. In addition, the S100A8 concentrations in serum and saliva showed significant differences according to the organ damage and were similar to the results of comparing the differences in S100 proteins depending on the presence of SDI scores in SLE patients with cardiovascular disease (31). The relationship between other clinical features and S100A8 levels in the serum, urine, and saliva was not consistent.

To the best of our knowledge, this is the first study to investigate the role of S100A8 protein as a diagnostic biomarker and its association with disease activity in patients with SLE using serum, urine, and saliva. It is important to analyze the S100 protein in the saliva of patients with SLE, which is a biofluid that is gradually receiving special attention, as it has been acknowledged that it contains many informative proteins about the disease process (44). Although the diagnostic ability of S100A8 that we have demonstrated is not superior to the diagnostic sensitivity and specificity of previously proposed classification criteria (45), it is worth emphasizing that we have discovered a powerful diagnostic biomarker in various biofluids. Due to the heterogeneous nature of SLE, a single diagnostic marker is not realistic; therefore, biofluid-based biomarkers will be indispensable in the future in terms of reliability, price, easy sampling, safety, and reproducibility. Another strength of our study is that the samples were collected in a consistent process by the same researcher using a cohort, and a prospective follow-up study under the same conditions for monitoring SLE disease activity is possible. Our cohort collects clinical information and biofluid samples of SLE patients annually; therefore, we will demonstrate the ability of S100A8 as a biomarker to predict the flare of SLE in future studies.

However, this study had some limitations. First, some of the samples included in the study may not have belonged to newly diagnosed patients; thus, concomitant medications such as immunosuppressants may have affected the concentration of S100A8. Second, a cross-sectional study using a cohort sample showed a difference in the number of samples obtained, depending on the type of biofluid, and the number of saliva samples was remarkably small. Finally, saliva biology and circadian variation may have affected the salivary samples, and only unstimulated saliva was collected. Future studies using unstimulated and stimulated saliva collected at the same time will be essential to verify our findings.

In summary, our study provides insights into the potential diagnostic role of S100A8 levels in the serum, urine, and saliva of patients with SLE. Serum, urine, and salivary S100A8 levels have good diagnostic ability, and a combination of various biofluids instead of a single biomarker will demonstrate their usefulness as a robust screening tool. Moreover, these biofluid-based biomarkers will be helpful indicators for monitoring SLE disease activity and predicting treatment response.

## Data Availability Statement

The original contributions presented in the study are included in the article/supplementary material. Further inquiries can be directed to the corresponding author.

## Ethics Statement

The studies involving human participants were reviewed and approved by the Institutional Review Board of Ajou University Hospital. The patients/participants provided their written informed consent to participate in this study.

## Author Contributions

J-WK acquired, analyzed, and interpreted the data and drafted the work. J-YJ, S-WL, W-YB, and H-AK analyzed and interpreted the data. C-HS conceptualized and designed the work, interpreted the data, and revised the manuscript. All authors approved the submitted version.

## Funding

This work was supported by a grant from the Korea Health Technology R&D Project (HR16C0001) through the Korea Health Industry Development Institute (KHIDI) funded by the Ministry of Health and Welfare.

## Conflict of Interest

The authors declare that the research was conducted in the absence of any commercial or financial relationships that could be construed as a potential conflict of interest.

## Publisher’s Note

All claims expressed in this article are solely those of the authors and do not necessarily represent those of their affiliated organizations, or those of the publisher, the editors and the reviewers. Any product that may be evaluated in this article, or claim that may be made by its manufacturer, is not guaranteed or endorsed by the publisher.

## Abbreviations

Ab, antibodies; aPL, antiphospholipid; AUC, area under the curve; dsDNA, double-stranded DNA; ESR, erythrocyte sedimentation rate; HCs, healthy controls; NPV, negative predictive value; PPV, positive predictive value; ROC, receiver operating characteristic; S100A8, S100 calcium-binding protein A8 protein; SLE, systemic lupus erythematosus; SLEDAI, SLE disease activity index; UPCR, urine protein/creatinine ratio.
